# Nitrergic and Substance P Immunoreactive Neurons in the Enteric Nervous System of the Bottlenose Dolphin (*Tursiops truncatus*) Intestine

**DOI:** 10.3390/ani11041057

**Published:** 2021-04-08

**Authors:** Cristiano Bombardi, Anna Maria Rambaldi, Giorgia Galiazzo, Fiorella Giancola, Jean-Marie Graïc, Giulia Salamanca, Bruno Cozzi, Roberto Chiocchetti

**Affiliations:** 1 Department of Veterinary Medical Sciences (UNI EN ISO 9001:2008), University of Bologna, 40064 Ozzano dell’Emilia, Bologna, Italy; cristiano.bombardi@unibo.it (C.B.); annamaria.rambaldi@gmail.com (A.M.R.); giorgia.galiazzo2@unibo.it (G.G.); fiorella.giancola2@unibo.it (F.G.); giulia.salamanca@studio.unibo.it (G.S.); 2 Unit of Veterinary Histology and Pathology, University Institute of Animal Health and Food Safety (IUSA), Veterinary School, University of Las Palmas de Gran Canaria, 35413 Las Palmas, Spain; 3 Department of Translational Medicine, University of Ferrara, 44121 Ferrara, Italy; 4 Department of Comparative Biomedicine and Food Science, University of Padova, 35020 Legnaro, Padova, Italy; jeanmarie.graic@unipd.it (J.-M.G.); bruno.cozzi@unipd.it (B.C.)

**Keywords:** cetaceans, gut, intestine, immunohistochemistry, nNOS

## Abstract

**Simple Summary:**

The gastrointestinal tract of the bottlenose dolphin (*Tursiops truncatus*) differs structurally and functionally from that of terrestrial mammals. In particular, the intestine does not show any macroscopic subdivision and lacks a caecum. In addition, the histological aspect of the intestine is relatively constant, without marked differences between the anterior and posterior parts. Although the intestine of these cetaceans presents differences in comparison with terrestrial mammals, little information is currently available on their enteric nervous system. The aim of the present study was to investigate the morphological and quantitative aspects of neurons immunoreactive (IR) for the neuronal nitric oxide synthase (nNOS) and Substance P (SP) in the intestine of bottlenose dolphins (*Tursiops truncatus*). In these dolphin specimens, a smaller number of nNOS-IR neurons in the submucosal plexus and a larger number of SP-IR neurons in the myenteric plexus were observed compared to other mammals. Interestingly, no co-localization between nNOS- and SP-IR neurons was detected in either of the plexuses, suggesting the existence of two completely distinct functional classes of neurons in the intestine of the bottlenose dolphin.

**Abstract:**

Compared with other mammals, the digestive system of cetaceans presents some remarkable anatomical and physiological differences. However, the neurochemical features of the enteric nervous system (ENS) in these animals have only been described in part. The present study gives a description of the nitrergic and selected peptidergic systems in the myenteric plexus (MP) and submucosal plexus (SMP) of the intestine of the bottlenose dolphin (*Tursiops truncatus*). The distribution and morphology of neurons immunoreactive (IR) for the neuronal nitric oxide synthase (nNOS) and Substance P (SP) were immunohistochemically studied in formalin-fixed specimens from the healthy intestine of three animals, and the data were compared with those described in the literature on other mammals (human and non-human). In bottlenose dolphins, the percentages of nitrergic neurons (expressed as median and interquartile range—IQR) were 28% (IQR = 19–29) in the MP and 1% (IQR = 0–2) in the SMP, while the percentages of SP-IR neurons were 31% (IQR = 22–37) in the MP and 41% (IQR = 24–63) in the SMP. Although morphological features of nNOS- and SP-IR neurons were similar to those reported in other mammals, we found some noticeable differences in the percentages of enteric neurons. In fact, we detected a lower proportion of nNOS-IR neurons in the SMP and a higher proportion of SP-IR neurons in the MP compared to other mammals. To the best of the authors’ knowledge, this study represents the first description and quantification of nNOS-IR neurons and the first quantification of SP-IR neurons in the intestine of a cetacean species. As nNOS and SP are important mediators of intestinal functions and the nitrergic population is an important target for many neuroenteropathies, data obtained from a healthy intestine provide a necessary basis to further investigate and understand possible functional differences and motor intestinal dysfunctions/alterations in these special mammals.

## 1. Introduction

Marine Cetartiodactyla underwent extensive morphological and physiological evolutionary adaptations to life in the water [1]. Salinity and wide variations of temperature and pressure are just a few of the environmental characteristics that cetaceans have to deal with. Like other organs and systems of odontocetes, the gastrointestinal (GI) tract differs structurally and functionally from that of terrestrial mammals. The tongue has no taste buds, except in certain species [2], prey is swallowed without being chewed and the larynx, which passes through the pharynx, can be voluntarily displaced to allow the passage of food [2,3]. The stomach of delphinids consists of multiple chambers, including a highly muscular forestomach, necessary to grind and digest the whole prey [4,5,6,7]. Unlike terrestrial Cetartiodactyla (including the closely related ruminants and *Hippopotamidae*) which also have multiple-chamber stomachs, the stomach complex of cetaceans does not promote multiple chewing cycles. The intestine itself does not show any macroscopic subdivision into small and large intestine, and the caecum is absent; in addition, the histological aspect is also relatively constant, without marked differences between the anterior and posterior parts [5,6,8]. Cetaceans also lack a gall bladder, an arguable evolutionary consequence of the continuous ingestion of food [6].

Although the digestive system of cetaceans presents such differences, when compared with terrestrial mammals, little information is currently available on their enteric nervous system (ENS) [8,9,10,11,12]. The ENS regulates the great majority of digestive functions and activities such as motility, absorption, secretion and blood flow [13]. In the last decade, advances in our understanding of the brain–gut axis have shown the tremendous influence of the ENS on immune, humoral and metabolic homeostasis (Kulkarni et al., 2018). It consists of a huge integrated network of neurons and fibers arranged in the wall of the digestive system, from the esophagus to the internal anal sphincter, and extending to the pancreas and extrahepatic biliary system [14,15]. In the ENS, neurons, fibers and enteric glial cells are organized into two major ganglionated plexuses, the myenteric (MP) and the submucosal plexus (SMP) [13]. The MP is located between the longitudinal (LML) and circular (CML) muscle layers, and provides motor innervation to the GI smooth muscle cells, while the SMP regulates mainly mucosal and submucosal functions and activities, at least in small laboratory rodents [15]. Enteric neurons can be grouped into different functional classes (intrinsic primary afferent neurons (IPANs), excitatory and inhibitory motor neurons and interneurons), based on their specific neurochemical code (i.e., the cocktail of neurotransmitters that they synthesize). Nitric oxide (NO) is the most important inhibitory neurotransmitter of the GI tract, and in most mammalian species, it is also synthesized by descending interneurons, which do not necessarily have an inhibitory action. Mostly released by MP neurons, NO induces relaxation of the GI musculature and sphincters by acting directly on the intestinal smooth muscle cells or by attenuating the release of the excitatory neurotransmitters, such as acetylcholine and substance P (SP) [16,17]. Nitric oxide is synthesized through the activation of neuronal nitric oxide synthase (nNOS), an enzyme that can be found in MP and SMP neurons and fibers of different species [18,19,20,21]. 

SP belongs to the tachykinin family, a group of neuropeptides involved not only in the regulation of different gastrointestinal functions, such as motility and secretion, but also in inflammation and pain genesis [22,23,24,25,26]. In the gut, SP is found in MP excitatory muscular motor neurons and MP and SMP IPANs, as well as in extrinsic sensory fibers and enteroendocrine cells [9,15,25]. Frequently detected with acetylcholine in intestinal intramural neurons and fibers, SP is considered a cholinergic co-mediator [15,27]. Since NO and SP play important roles in intestinal motor function, they have been widely regarded as relevant neurotransmitters in the study of GI motility disorders [28,29,30,31]. 

The aim of the present study was to describe and quantify nNOS and SP immunoreactive enteric neurons in the non-pathological gut of bottlenose dolphins. By doing so, we provide a first insight into the complex interaction between two major neurochemical classes of enteric neurons in the intestine of this common marine mammal.

## 2. Materials and Methods

### 2.1. Animals

Samples of intestine of three adult bottlenose dolphins were obtained from the Mediterranean marine mammal tissue bank of the Department of Comparative Biomedicine and Food Science of the University of Padova (Italy) (MMMTB, www.marinemammals.eu, accessed on 2 April 2021). The MMMTB (CITES IT020) works under the auspices of the Italian Ministry for the Environment and the University of Padova, and receives tissue specimens from cetaceans stranded along the Italian coast of the Mediterranean, or directly samples tissues from dolphins and whales brought to its facilities for post mortem diagnosis. 

According to Directive 2010/63/EU of the European Parliament and of the Council of 22 September 2010 regarding the protection of animals used for scientific purposes, the Italian legislation (D. Lgs. n. 26/2014) does not require any approval by competent authorities or ethics committees because this study did not influence any therapeutic decisions.

### 2.2. Tissue Collection

Tissues were collected from the antimesenteric side of the intestine in the putative jejunal portion caudal to the duodenal ampulla (marked by the pancreatic and hepatic ducts).

All carcasses were coded for freshness [32]. Once removed, tissue fragments were washed in PBS (0.1 M phosphate buffer saline, pH 7.4), and immersed in 4% buffered formalin for at least 24 h at 4 °C; following fixation, tissue samples were dehydrated and embedded in paraffin. Serial longitudinal and transverse sections (7 µm thick) were collected on poly-L-lysine-coated slides and processed for histological and immunohistochemical labeling.

### 2.3. Histology

One section for each specimen was stained with hematoxylin and eosin (H&E) to assess tissue condition. Microscopic analysis of the sections showed the absence of pathological alteration in the gut (data not shown), and therefore the tissues from all three animals were included in the present research.

### 2.4. Double Immunofluorescence 

To assess the proportion of subclasses of neurons and evaluate their co-localization, we performed double immunostaining with immunolabeling for either PGP9.5 and nNOS, PGP9.5 and SP or nNOS and SP. Briefly, sections were deparaffinized in xylene, rehydrated through graded ethanol and heated in sodium citrate buffer (pH 6.0) in a microwave (5 min at 700 W) for antigen retrieval. To block non-specific bindings, sections were incubated for 1.5 h at room temperature (RT) in a solution containing 20% normal goat serum (CS9022, Colorado Serum Co., Denver, CO, USA) or 20% normal donkey serum (D9663, Sigma-Aldrich, Saint Louis, MO, USA), and 0.5% Triton X-100 (Merck, Darmstadt, Germany) in PBS. Sections were then incubated overnight in a humid chamber at RT in a cocktail of primary antibodies (Table 1) diluted in 1.8% NaCl in 0.01 M PBS containing 0.1% sodium azide. After rinsing in PBS (3 × 10 min), the sections were incubated for 1.5 h at RT in a solution of secondary antibodies (Table 1) diluted in PBS. Enteric neurons were identified using blue fluorescent Nissl stain solution (NeuroTrace^®^, Molecular Probes, Eugene, OR, USA—NT throughout the text) for 90 min and/or the antibody guinea pig anti-PGP 9.5 (see Table 1 for details). The two neuronal markers identified the same neurons of the MP and SMP (Supplementary Figure S1). After washing, the sections were mounted in buffered glycerol at pH 8.6. 

### 2.5. Specificity of the Primary Antibodies 

The immunogen used to obtain antibody anti-nNOS (Ab5380) was the recombinant human neuronal nitric oxide synthase 1. The homology between the full amino acid sequences of *Tursiops truncatus* (A0A6J3PRR6_TURTR) and Human nNOS (P29475 NOS1_HUMAN) was 94.3%. 

The immunogen used to obtain antibody anti-SP (10-S15A) was substance-P-BSA. The sequence of SP is very well conserved among many mammalian species [34], including the *Tursiops truncatus* (A0A2U3V1Z_TURTR), which shows 96.1% homology with the full amino acid sequence of *Human* SP (P20366 SP_HUMAN) and the 100 % homology with the peptidic sequence used as immunogen (RPKPQQFFGLM). 

The homologies of nNOS and SP of *Tursiops truncatus* were verified by the “alignment” tool available on the Uniprot database (www.uniprot.org, accessed on 2 April 2021) and the BLAST tool of the National Center for Biotechnology information (NCBI) (www.ncbi.nlm.nih.gov, accessed on 2 April 2021). 

In addition, the anti-SP and anti-nNOS antibodies utilized in the present study have been already successfully employed in the nervous system of the dolphins [35,36].

### 2.6. Specificity of the Secondary Antibodies

The specificity of the secondary antibodies (Table 1) was tested by the absence of signal after the exclusion of the primary antibodies on bottlenose dolphin intestinal tissues.

### 2.7. Analysis of Sections

Immunohistochemical preparations were analyzed with a Nikon Eclipse Ni microscope equipped with the appropriate filter cubes. The images were recorded with a DS-Qi1Nc digital camera and NIS Elements software BR 4.20.01 (Nikon Instruments Europe BV, Amsterdam, The Netherlands). The proportions of neurons that were immunoreactive for nNOS or SP were determined by examining fluorescent double-stained preparations. Neurons were first located by PGP9.5 immunostaining and/or by the presence of a fluorophore that labelled NT and then the filter was switched to determine whether or not the neuron was labelled for a second antigen (nNOS or SP), located with a fluorophore of a different colour.

In this way, proportions of neurons labeled for pairs of antigens were determined. At least 200 NT-labeled neurons were counted for each sample tissue from each animal and the percentage of neurons that were NT labeled and/or PGP 9.5 immunolabeled and that were also immunoreactive for nNOS or SP was calculated and expressed as a relative percentage (median and interquartile range—IQR).

## 3. Results

### 3.1. Hematoxylin and Eosin Staining

In the bottlenose dolphin, the MP was organized in large ganglia located between the longitudinal (LML) and circular muscle layer (CML). In longitudinal and transverse sections, ganglia were different sizes and contained up to 43 neurons. The neurons of the SMP were organized in smaller ganglia (up to 18 neurons) distributed at two different levels within the submucosal layer. The inner submucosal plexus (ISMP) was composed of small ganglia located near the *muscularis mucosae,* harboring small cell bodies, whereas the outer submucosal plexus (OSMP), lying close to the CML, was composed of larger neurons. Solitary neurons were also observed, dispersed in the submucosal layer.

### 3.2. Nitrergic Neurons

**Figure 1:**
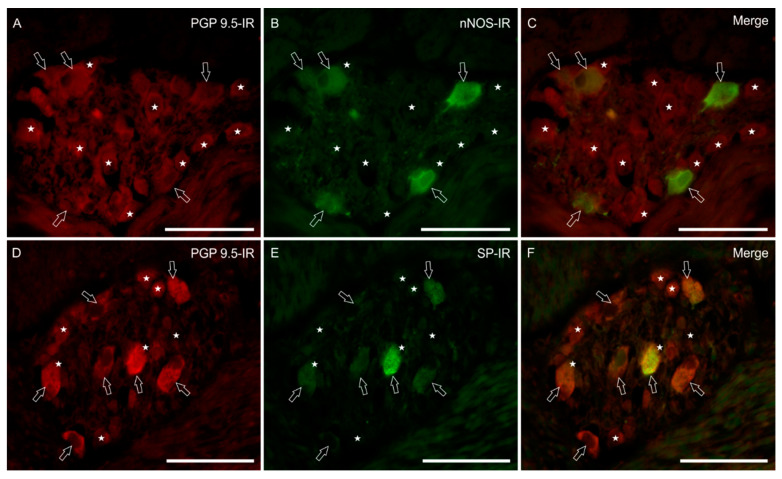
Micrographs showing nNOS (**A**–**C**) and SP immunoreactivity (IR) (**D**–**F**) in longitudinal sections of the myenteric plexus in bottlenose dolphin intestine. Stars indicate PGP 9.5 immunoreactive myenteric plexus neurons (**A**,**D**) which were not immunoreactive for the neuronal nitric oxide synthase (nNOS-IR) and substance P (SP-IR); arrows indicate PGP 9.5 immunoreactive neurons, which were also nNOS-IR (**B**) and SP-IR (**E**). (**C**,**F**) Merged images. Scale bar: 50 µm.

The nitrergic subpopulation of enteric neurons presented a homogeneous immunoreactivity of the soma, without nuclear labeling. These neurons showed an irregular outline and short processes, resembling Dogiel type I neurons. In the MP, nNOS immunoreactive (nNOS-IR) neurons represented 28% (IQR = 19–29) of the total neuronal population (404/1478 cells; *n* = 3) (Figure 1A–C). In the SMP, nNOS-IR neurons were observed just occasionally (1% IQR = 0–2), and only in the OSMP, close to the CML (9/1132 cells, *n* = 3) (data not shown).

### 3.3. SP-IR Neurons

**Figure 2:**
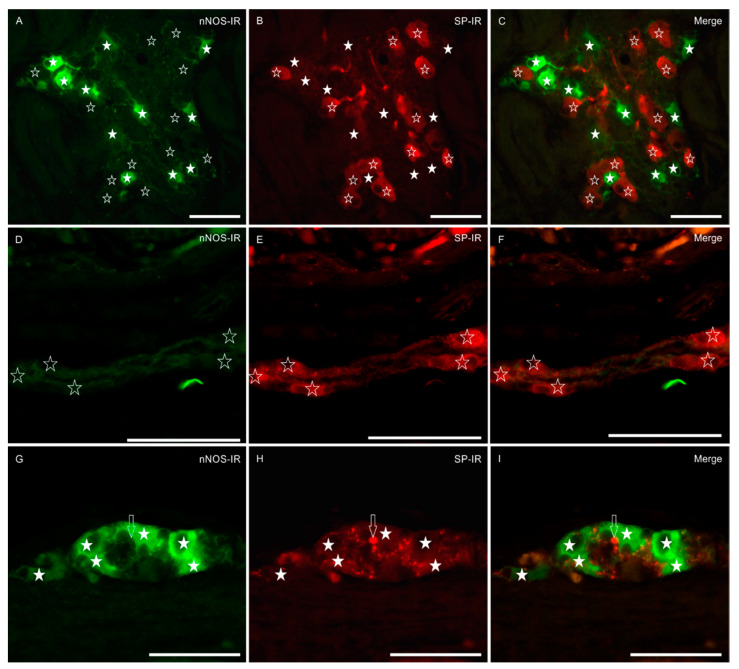
Micrographs of the sections of the myenteric plexus (MP) (**A**–**C**) and submucosal plexus (SMP) (**D**–**I**) of the bottlenose dolphin intestine. White stars indicate neurons showing neuronal nitric oxide synthase (nNOS) immunoreactivity (nNOS-IR); open stars indicate neurons showing substance P-IR (SP-IR). (**A**–**C**) Sections in which it is evident that no MP neurons co-expressed the two neuronal markers. (**D**–**I**) The SMP of the dolphin intestine was double layered; in the inner SMP (**D**–**F**), neurons preferentially expressed SP-IR (**E**) and were nNOS negative (**D**); nitrergic neurons were only observable in the outer SMP (**G**–**I**). The *arrows* indicate SP-IR varicosities encircling nitrergic neurons. (**C**,**F**,**I**) Merged images. Scale bar: 50 µm.

SP-IR was expressed by 31% (IQR = 22–37) of MP (456/1478 cells, *n* = 3) and 41% (IQR = 24–63) of SMP neurons (412/1132 cells, *n* = 3). The majority of SP-IR myenteric neurons showed a smooth outline of the cell body, a typical feature of Dogiel type II neurons (Figure 1D–F). In the myenteric neuropil, we frequently observed bright SP-IR nerve fibers forming baskets of SP-IR varicosities around nNOS-IR and nNOS-negative neurons (Figure 2G–I). Immunolabeled varicosities and fibers were frequently visible around submucosal blood vessels (data not shown).

### 3.4. Co-Localizations of nNOS and SP

No co-localization between the two markers was detected, in either of the plexuses (Figure 2A–I).

## 4. Discussion

One of the unsolved problems with the functional anatomy of the GI tract in dolphins and whales is its subdivision. Since the post-gastric intestine shows no macroscopic change of morphology and diameter, a classification into small and large intestine or any further subdivision is impossible at the moment [2,7]. Waiting for a specific study that may identify the branches of the mesenteric arteries, and thus establish a phylogenetic comparative criterion to adopt for the subdivisions of the cetacean gut, here we describe the whole system without further classification. Our observations on the histological architecture of the ENS in the intestine of the bottlenose dolphin confirm previous findings [5,8,9,10]. A multi-layered distribution of the submucosal ganglia has already been reported in the intestine of other large mammals [37,38,39,40,41,42,43,44,45,46,47,48,49].

Several neurotransmitters, including tachykinins and NO, regulate the contractility of the GI musculature. The balance between excitatory and inhibitory signals to smooth muscle cells generates all the intestinal physiological motor patterns. As NO is the main transmitter involved in inhibitory inputs and SP is considered a co-transmitter in cholinergic neurons, the present study provides the first comprehensive insight into these two functional classes of neurons in the intestine of the bottlenose dolphin. Our data on the nitrergic MP subpopulation (28%) are partially consistent with those obtained in other large terrestrial mammals [50,51,52,53,54,55,56] and small terrestrial mammals [15,57,58]. Studies on rats indicate that nitrergic neurons account for 23 to 30% of the entire neuronal population in the ileum [59,60,61,62,63] and for about 34% in the colon [63,64].

The percentage obtained in the MP of bottlenose dolphins diverges from data obtained in the small and large human intestine, where nitrergic neurons represent about 34% and 38%, respectively [65], and from data obtained in the porcine colon, where nitrergic neurons account for about 50% of the enteric neuronal population [66]. 

In the SMP, nNOS-IR was expressed only by 1% of neurons, exclusively located in the large ganglia close to the CML, whereas they were absent in the ISMP. These percentages are similar to those previously described in the horse ileum [52] and in the small intestine of pigs [55,67,68,69] where nNOS-IR neurons represented from 1% to 8% of the total SMP neurons. On the other hand, our percentages are quite different from those obtained in the lamb ileum [51] and pig ascending colon [66], where SMP nNOS-IR neurons account for 21% of the former and 45% of the latter. 

The great majority of nNOS-IR cells observed in the present research were Dogiel type I shaped. Dogiel type I cells, characterized by an angular outline of the soma and numerous dendritic processes, include interneurons and inhibitory motor neurons [15]. Interestingly, in the bottlenose dolphin intestine, we observed the presence of nitrergic neurons in the OSMP but not in the ISMP. In other species, it is well known that the ISMP and the OSMP present different neurochemical coding and different electrophysiological properties and, therefore, have distinct functions [45,68,70,71,72]. As OSMP neurons actually show a phenotype more similar to that of the myenteric plexus neurons, it is plausible that they supply an inhibitory innervation of the CML [73,74].

Several pathological conditions of the gastrointestinal tract of humans and other mammals are associated with an impairment of NO neurons [28,31,56,75,76,77,78,79,80,81,82]. Thus, to better understand the implications of nitrergic neurons in gastrointestinal pathologies of dolphins, it is essential to investigate, in particular, the architecture and neurochemistry of the ENS, especially its inhibitory components, in tissues of healthy animals.

To our knowledge, the distribution of SP-IR in the gastrointestinal tract of cetaceans was described only in the striped dolphin (*Stenella coeruleoalba*) by Domeneghini et al. [10], who reported generic values (i.e., low, average or high numbers) of intestinal SP-IR structures. In the bottlenose dolphin intestine, the myenteric SP-IR subpopulation accounts for 31% of the total neuronal population. This value is relatively higher than the percentages reported in the ileum of sheep (13%) [53], horses (14%) [52] and in the small intestine of pigs (from 1% to 9%) [55,69]. The majority of SP-IR neurons observed in the present study resemble Dogiel type II cells. Since SP-IR neurons could possibly be IPANs, interneurons or excitatory muscular motor neurons [15,83,84], it seems unlikely that almost all MP neurons were Dogiel type II cells, because motor neurons and interneurons usually show Dogiel type I morphology. One explanation might be a reduced immunostaining of SP-IR neurons with the appearance of a smooth outline. However, in contrast to this last hypothesis, there is the clear morphology of nNOS-IR neurons, which showed an unequivocal Dogiel type I morphology.

In the SMP of the bottlenose dolphin, SP-IR neurons represent 41% of the total neuronal population and were largely located in the ISMP. This percentage is similar to that described in the ileum of sheep (38%) [51,53] and in the small intestine of piglets (from 20% to 42%, depending on the tract) [55,69], whereas it differs significantly from data obtained from the horse ileum [52] and from the colon of piglets [85], where SP-IR neurons correspond to 66% and 87% of the total neuronal population, respectively. 

As reported in other mammals, like the striped dolphin [10] and the horse [52], we also observed baskets of SP-IR varicosities and fibers around the MP nitrergic and non-nitrergic neurons in the bottlenose dolphin. These fibers, located around somata, might arise from IPANs and interneurons [86] or from peripheral processes of dorsal root ganglion afferent neurons, which participate in the control of gastrointestinal activities [87]. Puzzling and hitherto unconfirmed findings in other marine Cetartiodactyla belonging to the *Ziphiidae* and *Delphinapteridae* families (Pfeiffer, 1993) reported a modified innervation of the myenteric plexus and changes in the *muscularis externa*. Specifically, the musculature of the gut showed the presence of intercalation-like striations, thus hinting at the possibility of a more specific control the movements of the gut, presumably functional in the peculiar modality of suction feeding [2,9]. The presence and distribution of visceral nitrergic and substance P immunoreactive neurons that we report here may further support the existence of such physiological mechanisms; however, voluntary movements would most likely require different neurochemical profiles which would need further investigation.

In contrast to what was described in sheep [53] and similarly to what was described in the small intestine of the mouse [57], no co-localization between nNOS- and SP-IR was detected in either the plexuses, suggesting the existence of two completely distinct functional classes of neurons in the intestine of the bottlenose dolphin. Indeed, also in colon, recent studies have shown distinctly segregated excitatory and inhibitory neurons [88], and their synaptic connections via intrinsic sensory neurons [88].

### Limitations of the Study

This study should be considered as an initial preliminary overview and description of nNOS- and SP-IR neurons in the intestine of bottlenose dolphins. Despite many efforts to make the study scientifically accurate, we are aware of the limitations intrinsic to the present research. First of all are the disadvantages of studying free-range animals. In fact, the time between death and sampling is not always as short as it should be, especially regarding tissue in direct contact with the animal’s microbiota. To avoid this problem, we chose animals based on the conservation grade that they were assigned, and we selected samples only from the freshest carcasses, with a conservation grade of 1 or 2 [31]. Nevertheless, among the animals chosen, a difference in the quality and preservation of the tissues could have existed, and it could have affected the immunoreactive properties. Furthermore, as there are fewer studies of cetaceans than there are of domestic animals, many antibodies were found to be useless. For instance, after various unsuccessful attempts with antibodies against choline acetyltransferase (CHAT), we were forced to use SP to mark excitatory neurons. In the same way, after many fruitless attempts with the pan-neuronal marker HuC/HuD, we decided to use NT and PGP9.5 in parallel in order to identify neurons in both plexuses. In the abovementioned cases, the lack of immunostaining could reflect conformational differences of the proteins in dolphins or be the consequence of bad tissue preservation. Another limitation was the reduced number of bottlenose dolphin included in the study. For the abovementioned reasons, the results reported here, although of interest, need to be supported by further investigation, with a larger number of cases.

At present, we are unaware of any other quantitative data obtained from other cetaceans with which to compare our data on the bottlenose dolphin. It is important to acknowledge major difference in diet and environment between marine and terrestrial mammals.

## 5. Conclusions

This is, to the best of the authors’ knowledge, the first description and quantification of nNOS-IR neurons, and the first quantification of SP-IR neurons as well, in the intestine of a cetacean species.

Although the general characteristics and morphology of nNOS- and SP-IR neurons are conserved among most mammal species, we found differences in the relative prevalence of neurons expressing either markers, consisting mainly in a very small number of nNOS-IR neurons in the SMP, and a larger number of MP SP-IR neurons. Further investigation is needed to identify neurochemical classes of neurons and fibers in order to give a clearer and more comprehensive picture of the ENS complexity in this species. Providing information on the physiological conditions of a healthy intestine, including its nervous component, is crucial to the understanding of its pathological states.

## Figures and Tables

**Table 1:** Details of antibodies and NeuroTrace^®^ used.

Primary Antibodies and NT	Host	Code	Dilution	Source
CHAT	Goat	Ab 144P	1:25	Millipore
cCHAT	Rabbit		1:100	Generous gift of Dr. K. Lips ^a^
pCHAT	Rabbit		1:1000	Generous gift of Prof Kimura ^b^
HuC/HuD	Mouse	A21271	1:200	Molecular Probes
HuC	Goat	SC-5977	1:100	Santa Cruz Biotechnologies
nNOS	Rabbit	Ab5380	1:300	Millipore
PGP 9.5	Guinea pig	Ab5898	1:100	Millipore
SP	Rat	10-S15A	1:300	Fitzgerald
NT		N21479	1:200	Molecular Probes
Secondary Antibodies	Dilution		Source	
Goat anti-rabbit IgG FITC	1:200		Calbiochem	
Donkey anti-rat IgG Alexa 594	1:50		Invitrogen	
Goat anti-guinea pig TRITC	1:100		Chemicon	

Abbreviations: CHAT, choline acetyltransferase; cCHAT, central choline acetyltransferase; pCHAT, peripheral choline acetyltransferase; nNOS, neuronal nitric oxide synthase; NT, blue fluorescent Nissl stain solution; PGP 9.5, protein gene product 9.5; SP, substance P; Suppliers: Calbiochem, Merck Millipore, Merck KGaA, Darmstadt, Germany; Chemicon, Merck Millipore, Merck KGaA, Darmstadt, Germany; Fitzgerald Industries International, Acton, Massachusetts, USA; Invitrogen, Thermo Fisher Scientific, Carlsbad, California, USA; Merck Millipore, Merck KGaA, Darmstadt, Germany; Molecular Probes, Eugene, Oregon, USA; Santa Cruz Biotechnologies, Dallas, Texas, USA; ^a^ Justus-Liebig-576 University, Giessen, Germany; ^b^ Shiga University of Medical Science, Otsu, Japan [33].

## Data Availability

The data presented in this study are available on request from the corresponding author.

## Supplementary Materials

The following are available online at https://www.mdpi.com/article/10.3390/ani11041057/s1, Figure S1: PGP 9.5 immunoreactivity in the myenteric plexus of the bottlenose dolphin intestine.

## References

[1] Reidenberg J.S. (2007). Anatomical adaptations of aquatic mammals. Anat. Rec..

[2] Cozzi B., Huggenberger S., Oelschläger H. (2017). Anatomy of Dolphins. Insights into Body Structure and Function.

[3] Reidenberg J.S., Laitman J.T. (1987). Position of the larynx in odontoceti (toothed whales). Anat. Rec..

[4] Harrison R.J., Johnson F.R., Young B.A. (1970). The oesophagus and stomach of dolphins (*Tursiops, Delphinus, Stenella*). J. Zool. Lond..

[5] Gaskin D.E. (1978). Form and function in the digestive tract and associated organs in Cetacea, with a consideration of metabolic rates and specific energy budgets. Oceanogr. Mar. Biol. Ann. Rev..

[6] Mead J.G., Perrin W., Wursing B., Thewissen J. (2008). Gastrointestinal tract. Encyclopedia of Marine Mammals.

[7] Huggenberger S., Oelschläger H., Cozzi B. (2019). Atlas of the Anatomy of Dolphins and Whales.

[8] Russo F., Gatta C., De Girolamo P., Cozzi B., Giurisato M., Lucini C., Varricchio E. (2012). Expression and immunohistochemical detection of leptin-like peptide in the gastrointestinal tract of the South American sea lion (*Otaria flavescens*) and the bottlenose dolphin (*Tursiops truncatus*). Anat. Rec..

[9] Pfeiffer C.J. (1993). Neural and muscular control functions of the gut in odontocetes: Morphologic evidence in beaked whales and beluga whales. J. Physiol. Paris.

[10] Domeneghini C., Massoletti P., Arrighi S. (1997). Localization of regulatory peptides in the gastrointestinal tract of the striped dolphin, *Stenella coeruleoalba* (*Mammalia: Cetacea*). An immunohistochemical study. Eur. J. Histochem..

[11] Naka T., Katsumata E., Sasaki K., Minamino N., Yoshioka M., Takei Y. (2007). Natriuretic peptides in cetacean: Identification, molecular characterization and changes in plasma concentration after landing. Zool. Sci..

[12] Gatta C., Russo F., Russolillo M.G., Varricchio E., Paolucci M., Castaldo L., Lucini C., de Girolamo P., Cozzi B., Maruccio L. (2014). The orexin system in the enteric nervous system of the bottlenose dolphin (*Tursiops truncatus*). PLoS ONE.

[13] Spencer N.J., Hu H. (2020). Enteric nervous system: Sensory transduction, neural circuits and gastrointestinal motility. Nat. Rev. Gastroenterol. Hepatol..

[14] Grundy D., Schemann M. (2005). Enteric nervous system. Curr. Opin. Gastroenterol..

[15] Furness J.B. (2006). The Enteric Nervous System.

[16] Sanders K.M., Ward S.M. (1992). Nitric oxide as a mediator of nonadrenergic noncholinergic neurotransmission. Am. J. Physiol..

[17] Stark M.E., Bauer A.J., Sarr M.G., Szurszewski J.H. (1993). Nitric oxide mediates inhibitory nerve input in human and canine jejunum. Gastroenterology.

[18] Costa M., Furness J.B., Pompolo S., Brookes S.J., Bornstein J.C., Bredt D.S., Snyder S.H. (1992). Projections and chemical coding of neurons with immunoreactivity for nitric oxide synthase in the guinea-pig small intestine. Neurosci. Lett..

[19] Ekblad E., Mulder H., Uddman R., Sundler F. (1994). NOS-containing neurons in the rat gut and coeliac ganglia. Neuropharmacology.

[20] Timmermans J.P., Barbiers M., Scheuermann D.W., Bogers J.J., Adriaensen D., Fekete E., Mayer B., Van Marck E.A., De Groodt-Lasseel M.H. (1994). Nitric oxide synthase immunoreactivity in the enteric nervous system of the developing human digestive tract. Cell Tissue Res..

[21] Timmermans J.P., Barbiers M., Scheuermann D.W., Stach W., Adriaensen D., Mayer B., De Groodt-Lasseel M.H. (1994). Distribution pattern, neurochemical features and projections of nitrergic neurons in the pig small intestine. Ann. Anat..

[22] Holzer P., Holzer-Petsche U. (1997). Tachykinins in the gut. Part I. Expression, release and motor function. Pharmacol. Ther..

[23] Holzer P., Holzer-Petsche U. (1997). Tachykinins in the gut. Part II. Roles in neural excitation, secretion and inflammation. Pharmacol. Ther..

[24] Maggi C.A., Catalioto R.M., Criscuoli M., Cucchi P., Giuliani S., Lecci A., Lippi A., Meini S., Patacchini R., Renzetti A.R. (1997). Tachykinin receptors and intestinal motility. Can. J. Physiol. Pharmacol..

[25] Shimizu Y., Matsuyama H., Shiina T., Takewaki T., Furness J.B. (2008). Tachykinins and their functions in the gastrointestinal tract. Cell. Mol. Life Sci..

[26] Steinhoff M.S., von Mentzer B., Geppetti P., Pothoulakis C., Bunnett N.W. (2014). Tachykinins and their receptors: Contributions to physiological control and the mechanisms of disease. Physiol. Rev..

[27] Brookes S.J. (2001). Classes of enteric nerve cells in the guinea-pig small intestine. Anat. Rec..

[28] Sivarao D.V., Mashimo H., Goyal R.K. (2008). Pyloric sphincter dysfunction in nNOS-/- and W/Wv mutant mice: Animal models of gastroparesis and duodenogastric reflux. Gastroenterology.

[29] King S.K., Sutcliffe J.R., Ong S.Y., Lee M., Koh T.L., Wong S.Q., Farmer P.J., Peck C.J., Stanton M.P., Keck J. (2010). Substance P and vasoactive intestinal peptide are reduced in right transverse colon in pediatric slow-transit constipation. Neurogastroenterol. Motil..

[30] Cellini J., Pommier R., Porter R., LePard K.J. (2012). Enhanced nerve-stimulated muscarinic and neurokinin contractions of ileum from streptozotocin guinea-pigs. Auton. Autacoid. Pharmacol..

[31] Masaoka T., Vanuytsel T., Vanormelingen C., Kindt S., Salim Rasoel S., Boesmans W., De Hertogh G., Farre R., Vanden Berghe P., Tack J. (2014). A spontaneous animal model of intestinal dysmotility evoked by inflammatory nitrergic dysfunction. PLoS ONE.

[32] Kuiken T., García-Hartmann M. Cetacean Dissection techniques and tissue sampling. Proceedings of the First ECS Workshop on Cetacean Pathology.

[33] Tooyama I., Kimura H. (2000). A protein encoded by an alternative splice variant of choline acetyltransferase mRNA is localized preferentially in peripheral nerve cells and fibers. J. Chem. Neuroanat..

[34] Holmgren S., Jensen J. (2001). Evolution of vertebrate neuropeptides. Brain Res. Bull..

[35] Bombardi C., Cozzi B., Nenzi A., Mazzariol S., Grandis A. (2011). Distribution of nitrergic neurons in the dorsal root ganglia of the bottlenose dolphin (*Tursiops truncatus*). Anat. Rec..

[36] Ramírez T., Sacchini S., Paz Y., Rosales R.S., Câmara N., Andrada M., Arbelo M., Fernández A. (2020). Comparison of Methods for the Histological Evaluation of Odontocete Spiral Ganglion Cells. Animals.

[37] Schabadasch A. (1930). Intramurale Nervengeflechte des Darmrohrs. Z. Zellforsch. Mikr. Anat..

[38] Gunn M. (1968). Histological and histochemical observations on the myenteric and submucous plexuses of mammals. J. Anat..

[39] Stach W. (1977). The external submucous plexus (Schabadasch) in the small intestine of the swine. I. Form, structure and connections of ganglia and nerve cells. Z. Mikrosk. Anat. Forsch..

[40] Christensen J., Rick G.A. (1987). Intrinsic nerves in the mammalian colon: Confirmation of a plexus at the circular muscle-submucosal interface. J. Auton. Nerv. Syst..

[41] Scheuermann D.W., Stach W., Timmermans J.P. (1987). Topography, architecture and structure of the plexus submucosus externus (Schabadasch) of the porcine small intestine in scanning electron microscopy. Acta Anat..

[42] Scheuermann D.W., Stach W., Timmermans J.P. (1987). Topography, architecture and structure of the plexus submucosus internus (Meissner) of the porcine small intestine in scanning electron microscopy. Acta Anat..

[43] Timmermans J.P., Scheuermann D.W., Stach W., Adriaensen D., De Groodt-Lasseel M.H. (1992). Functional morphology of the enteric nervous system with special reference to large mammals. Eur. J. Morphol..

[44] Timmermans J.P., Adriaensen D., Cornelissen W., Scheuermann D.W. (1997). Structural organization and neuropeptide distribution in the mammalian enteric nervous system, with special attention to those components involved in mucosal reflexes. Comp. Biochem. Physiol. A Physiol..

[45] Timmermans J.P., Hens J., Adriaensen D. (2001). Outer submucous plexus: An intrinsic nerve network involved in both secretory and motility processes in the intestine of large mammals and humans. Anat. Rec..

[46] Pearson G.T. (1994). Structural organization and neuropeptide distributions in the equine enteric nervous system: An immunohistochemical study using whole-mount preparations from the small intestine. Cell Tissue Res..

[47] Pompolo S. (1994). An immunohistochemical study of neuropeptides and neuron-specific proteins present in the small intestine of the black-capped capuchin (*Cebus appela*). Neurogastroenterol. Mot..

[48] Balemba O.B., Grondahl M.L., Mbassa G.K., Semuguruka W.D., Hay-Smith A., Skadhauge E., Dantzer V. (1998). The organisation of the enteric nervous system in the submucous and mucous layers of the small intestine of the pig studied by VIP and neurofilament protein immunohistochemistry. J. Anat..

[49] Balemba O.B., Mbassa G.K., Semuguruka W.D., Assey R.J., Kahwa C.K., Hay-Schmidt A., Dantzer V. (1999). The topography, architecture and structure of the enteric nervous system in the jejunum and ileum of cattle. J. Anat..

[50] Brehmer A., Schrodl F., Neuhuber W., Tooyama I., Kimura H. (2004). Co-expression pattern of neuronal nitric oxide synthase and two variants of choline acetyltransferase in myenteric neurons of porcine ileum. J. Chem. Neuroanat..

[51] Chiocchetti R., Grandis A., Bombardi C., Lucchi M.L., Dal Lago D.T., Bortolami R., Furness J.B. (2006). Extrinsic and intrinsic sources of calcitonin gene-related peptide immunoreactivity in the lamb ileum: A morphometric and neurochemical investigation. Cell. Tissue Res..

[52] Chiocchetti R., Bombardi C., Mongardi-Fantaguzzi C., Venturelli E., Russo D., Spadari A., Montoneri C., Romagnoli N., Grandis A. (2009). Intrinsic innervation of the horse ileum. Res. Vet. Sci..

[53] Mazzuoli G., Mazzoni M., Albanese V., Clavenzani P., Lalatta-Costerbosa G., Lucchi M.L., Furness J.B., Chiocchetti R. (2007). Morphology and neurochemistry of descending and ascending myenteric plexus neurons of sheep ileum. Anat. Rec..

[54] Freytag C., Seeger J., Siegemund T., Grosche J., Grosche A., Freeman D.E., Schusser G.F., Hartig W. (2008). Immunohistochemical characterization and quantitative analysis of neurons in the myenteric plexus of the equine intestine. Brain Res..

[55] Zacharko-Siembida A., Valverde Piedra J.L., Szymanczyk S., Arciszewski M.B. (2013). Immunolocalization of NOS, VIP, galanin and SP in the small intestine of suckling pigs treated with red kidney bean (Phaseolus vulgaris) lectin. Acta Histochem..

[56] Giancola F., Fracassi F., Gallucci A., Sadeghinezhad J., Polidoro G., Zini E., Asti M., Chiocchetti R. (2016). Quantification of nitrergic neurons in the myenteric plexus of gastric antrum and ileum of healthy and diabetic dogs. Auton. Neurosci..

[57] Qu Z.D., Thacker M., Castelucci P., Bagyanszki M., Epstein M.L., Furness J.B. (2008). Immunohistochemical analysis of neuron types in the mouse small intestine. Cell Tissue Res..

[58] Lawson V.A., Furness J.B., Klemm H.M., Pontell L., Chan E., Hill A.F., Chiocchetti R. (2010). The brain to gut pathway: A possible route of prion transmission. Gut.

[59] Nichols K., Staines W., Krantis A. (1993). Nitric oxide synthase distribution in the rat intestine: A histochemical analysis. Gastroenterology.

[60] Lin Z., Liu Y., Zheng Q., Hu Q. (2011). Increased proportion of nitric oxide synthase immunoreactive neurons in rat ileal myenteric ganglia after severe acute pancreatitis. BMC Gastroenterol..

[61] Mann P.T., Furness J.B., Southwell B.R. (1999). Choline acetyltransferase immunoreactivity of putative intrinsic primary afferent neurons in the rat ileum. Cell Tissue Res..

[62] Brasileiro A.D., Garcia L.P., de Carvalho da Silva S., Rocha L.B., Pedrosa A.L., Vieira A.S., da Silva V.J.D., Rodrigues A.R.A. (2019). Effects of diabetes mellitus on myenteric neuronal density and sodium channel expression in the rat ileum. Brain Res..

[63] Chiocchetti R., Hitrec T., Giancola F., Sadeghinezhad J., Squarcio F., Galiazzo G., Piscitiello E., De Silva M., Cerri M., Amici R. (2021). Phosphorylated Tau protein in the myenteric plexus of the ileum and colon of normothermic rats and during synthetic torpor. Cell Tissue Res..

[64] da Silva M.V., Marosti A.R., Mendes C.E., Palombit K., Castelucci P. (2015). Differential effects of experimental ulcerative colitis on P2X7 receptor expression in enteric neurons. Histochem. Cell Biol..

[65] Brehmer A., Schrodl F., Neuhuber W. (2006). Morphology of VIP/nNOS-immunoreactive myenteric neurons in the human gut. Histochem. Cell. Biol..

[66] Mazzoni M., Caremoli F., Cabanillas L., de Los Santos J., Million M., Larauche M., Clavenzani P., De Giorgio R., Sternini C. (2020). Quantitative analysis of enteric neurons containing choline acetyltransferase and nitric oxide synthase immunoreactivities in the submucosal and myenteric plexuses of the porcine colon. Cell Tissue Res..

[67] Timmermans J.P., Scheuermann D.W., Stach W., Adriaensen D., De Groodt-Lasseel M.H., Polak J.M. (1989). Neuromedin U-immunoreactivity in the nervous system of the small intestine of the pig and its coexistence with substance P and CGRP. Cell Tissue Res..

[68] Timmermans J.P., Scheuermann D.W., Stach W., Adriaensen D., De Groodt-Lasseel M.H. (1990). Distinct distribution of CGRP-, enkephalin-, galanin-, neuromedin U-, neuropeptide Y-, somatostatin-, substance P-, VIP- and serotonin-containing neurons in the two submucosal ganglionic neural networks of the porcine small intestine. Cell Tissue Res..

[69] Czajkowska M., Całka J. (2020). Neurochemistry of Enteric Neurons Following Prolonged Indomethacin Administration in the Porcine Duodenum. Front. Pharmacol..

[70] Crowe R., Kamm M.A., Burnstock G., Lennard-Jones J.E. (1992). Peptide-containing neurons in different regions of the submucous plexus of human sigmoid colon. Gastroenterology.

[71] Thomsen L., Pearson G.T., Larsen E.H., Skadhauge E. (1997). Electrophysiological properties of neurones in the internal and external submucous plexuses of newborn pig small intestine. J. Physiol..

[72] Thomsen L., Pearson G.T., Skadhauge E. (1997). Electrophysiological classification of submucosal plexus neurones in the jejunum of the newborn pig. Comp. Biochem. Physiol. A Physiol..

[73] Scheuermann D.W., Stach W., Timmermans J.P. (1988). Morphology and immunocytochemistry of the enteric nervous system in the porcine small intestine. Part II. Acta Gastroenterol. Belg..

[74] Hens J., Schrödl F., Brehmer A., Adriaensen D., Neuhuber W., Scheuermann D.W., Schemann M., Timmermans J.-P. (2000). Mucosal projections of enteric neurons in the porcine small intestine. J. Comp. Neurol..

[75] Vanderwinden J.M., De Laet M.H., Schiffmann S.N., Mailleux P., Lowenstein C.J., Snyder S.H., Vanderhaeghen J.J. (1993). Nitric oxide synthase distribution in the enteric nervous system of Hirschsprung’s disease. Gastroenterology.

[76] Bealer J.F., Natuzzi E.S., Flake A.W., Adzick N.S., Harrison M.R. (1994). Effect of nitric oxide on the colonic smooth muscle of patients with Hirschsprung’s disease. J. Pediatr. Surg..

[77] Tomita R., Munakata K., Kurosu Y., Tanjoh K. (1995). A role of nitric oxide in Hirschsprung’s disease. J. Pediatr. Surg..

[78] Takahashi T., Nakamura K., Itoh H., Sima A.A., Owyang C. (1997). Impaired expression of nitric oxide synthase in the gastric myenteric plexus of spontaneously diabetic rats. Gastroenterology.

[79] Ribeiro U., Safatle-Ribeiro A.V., Habr-Gama A., Gama-Rodrigues J.J., Sohn J., Reynolds J.C. (1998). Effect of Chagas’ disease on nitric oxide-containing neurons in severely affected and unaffected intestine. Dis. Colon Rectum.

[80] Spangeus A., Suhr O., El-Salhy M. (2000). Diabetic state affects the innervation of gut in an animal model of human type 1 diabetes. Histol. Histopathol..

[81] Takahashi T. (2003). Pathophysiological significance of neuronal nitric oxide synthase in the gastrointestinal tract. J. Gastroenterol..

[82] Rivera L.R., Poole D.P., Thacker M., Furness J.B. (2011). The involvement of nitric oxide synthase neurons in enteric neuropathies. Neurogastroenterol. Motil..

[83] Sang Q., Williamson S., Young H.M. (1997). Projections of chemically identified myenteric neurons of the small and large intestine of the mouse. J. Anat..

[84] Clerc N., Furness J.B., Li Z.S., Bornstein J.C., Kunze W.A. (1998). Morphological and immunohistochemical identification of neurons and their targets in the guinea-pig duodenum. Neuroscience.

[85] Petto C., Gabel G., Pfannkuche H. (2015). Architecture and Chemical Coding of the Inner and Outer Submucous Plexus in the Colon of Piglets. PLoS ONE.

[86] Furness J.B., Jones C., Nurgali K., Clerc N. (2004). Intrinsic primary afferent neurons and nerve circuits within the intestine. Prog. Neurobiol..

[87] Holzer P. (2007). Role of visceral afferent neurons in mucosal inflammation and defense. Curr. Opin. Pharmacol..

[88] Smolilo D.J., Costa M., Hibberd T.J., Brookes S.J.H., Wattchow D.A., Spencer N.J. (2019). Distribution, projections, and association with calbindin baskets of motor neurons, interneurons, and sensory neurons in guinea-pig distal colon. J. Comp. Neurol..

