# Supplementary figures and images for: Nitrergic and Substance P Immunoreactive Neurons in the Enteric Nervous System of the Bottlenose Dolphin (Tursiops truncatus) Intestine
Source: Animals (Basel). 2021 Apr 8;11(4):1057. doi: 10.3390/ani11041057 (PMC8069003; doi:10.3390/ani11041057)

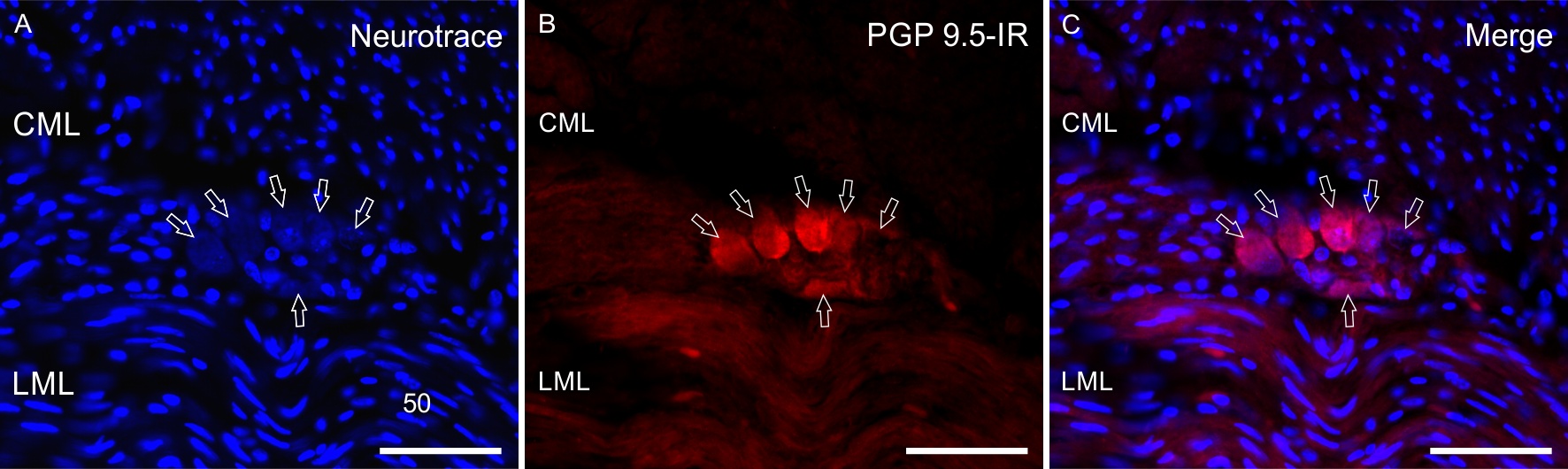

Supplement: Supplementary file 1 [file animals-11-01057-s001.zip › Fig. Suppl zippata/Figure S1.tif]
